# Cross-National Differences in Association Between Food Deprivation and Depressive Symptom in India, Mexico and USA

**DOI:** 10.1093/geroni/igaf122.646

**Published:** 2025-12-31

**Authors:** Shekhar Chauhan, Dawn Carr, T Muhammad, Arun Balachandran

**Affiliations:** Florida State University, Tallahassee, Florida, United States; Florida State University, Tallahassee, Florida, United States; The Pennsylvania State University, University Park, Pennsylvania, United States; Columbia University, New York City, New York, United States

## Abstract

Food insecurity has been shown to erode mental health as people age, but it is not known how these effects vary across different country contexts. This study examines the association between food deprivation and depressive symptoms among older adults in India, Mexico, and the USA. We use a nationally representative dataset we harmonized using Longitudinal Aging Study in India (LASI, 2017-18), the Mexican Health and Aging Study (MHAS, 2018), and the Health and Retirement Study (HRS, 2018). Descriptive analyses show differences in rates of food deprivation (5% in the USA, 7% in India, and 12% in Mexico) and depressive symptoms (4-item CESD) (0.66 symptoms in the US, 0.95 symptoms in India, and 1.13 symptoms in Mexico, on average) by country. OLS regression shows food deprivation is predictive of higher levels of depressive symptoms for older adults in all three countries relative to their non-food deprived counterparts. Moderation tests showed certain sociodemographic factors and resources differentially influence depressive symptom consequences of food deprivation in certain countries, but not others. For instance, females have higher depressive symptoms relative to their male counterparts only in Mexico (1.86 versus 1.25 symptoms; p < 0.001). Having higher levels of educational attainment is protective of food deprivation only in Mexico (p < 0.001). Living with children is protective only in India (1.3 vs 1.61; p < 0.001). However, being in worse health and being widowed is associated with higher depressive symptom consequences of food deprivation in all three countries (p < 0.001). These findings highlight the need for targeted policy interventions.

